# Acute right ventricular failure evoked by trauma induced thyroid storm supported by extracorporeal membrane oxygenation: A case report

**DOI:** 10.1097/MD.0000000000029359

**Published:** 2022-08-12

**Authors:** Soo Jin Park, Do Jung Kim, You Sun Hong, Sang Hyun Lim, Jiye Park

**Affiliations:** a Department of Thoracic and Cardiovascular Surgery, Ajou University School of Medicine, Suwon, Korea; b Division of Trauma Surgery, Department of Surgery, Ajou University School of Medicine, Suwon, Korea.

**Keywords:** acute heart failure, extracorporeal membrane oxygenation, thyroid storm, trauma

## Abstract

**Rationale:**

Cardiac arrest due to thyroid storm is a very rare clinical feature with high mortality that presents as multiorgan dysfunction. The mortality rate under this condition is close to 30%, even with appropriate treatment. Most thyroid storms occur in patients with long-standing untreated hyperthyroidism.

**Patient concerns:**

A 67-year-old woman, who had no specific medical history, was admitted with stupor mentality after a pedestrian traffic accident.

**Diagnosis:**

The patient had a Burch and Wartofsky score of 80, well beyond the criteria for diagnosis of a thyroid storm (>45 points).

**Interventions:**

Venoarterial extracorporeal membrane oxygenation (ECMO) was performed due to persistent unstable vital signs and findings of right ventricular dysfunction after return of spontaneous circulation after cardiopulmonary resuscitation. Circulatory assist with ECMO was performed for 8 days using a beta blocker, steroids, thionamide, and Lugol iodine solution.

**Outcomes:**

Myocardial function and thyroid hormone levels were rapidly normalized. The patient’s mental state recovered, and patient was discharged on day 36 maintaining medication.

**Lessons:**

Diagnosis of a thyroid storm in patients with multiple trauma is very difficult, because most trauma patients have symptoms of tachycardia, altered mental status, and abdominal pain that appear in thyrotoxic events. However, when unexplained shock without bleeding evidence occurs in patients with multiple trauma, a thyroid function test should be performed to rule out thyroid storm. Moreover, if hyperthyroidism is observed in a trauma patient, even if there is no history of hyperthyroidism, the possibility of a thyroid storm must be considered along with medical support treatment such as ECMO in patient with cardiogenic shock.

## 1. Introduction

A thyroid storm is a rare but fatal clinical disorder caused by thyrotoxicosis^[1,2]^ that presents as multiorgan dysfunction. The mortality rate of this condition is close to 30%, even with appropriate treatment.^[3]^ Most thyroid storms occur in patients with long-standing untreated hyperthyroidism.^[4–6]^ Trauma can trigger thyroid storms but only rarely.^[7]^ Venoarterial extracorporeal membrane oxygenation(ECMO) can provide temporary mechanical circulatory support for acute cardiopulmonary collapse.^[8]^ Since the concept of ECMO was first introduced in 1972, its use worldwide for refractory cardiogenic shock has been common.^[9,10]^ Here, we report the clinical usefulness of ECMO support in acute heart failure due to a thyroid storm after trauma in a patient with no specific endocrine history.

## 2. Case presentation

**Figure 1.**
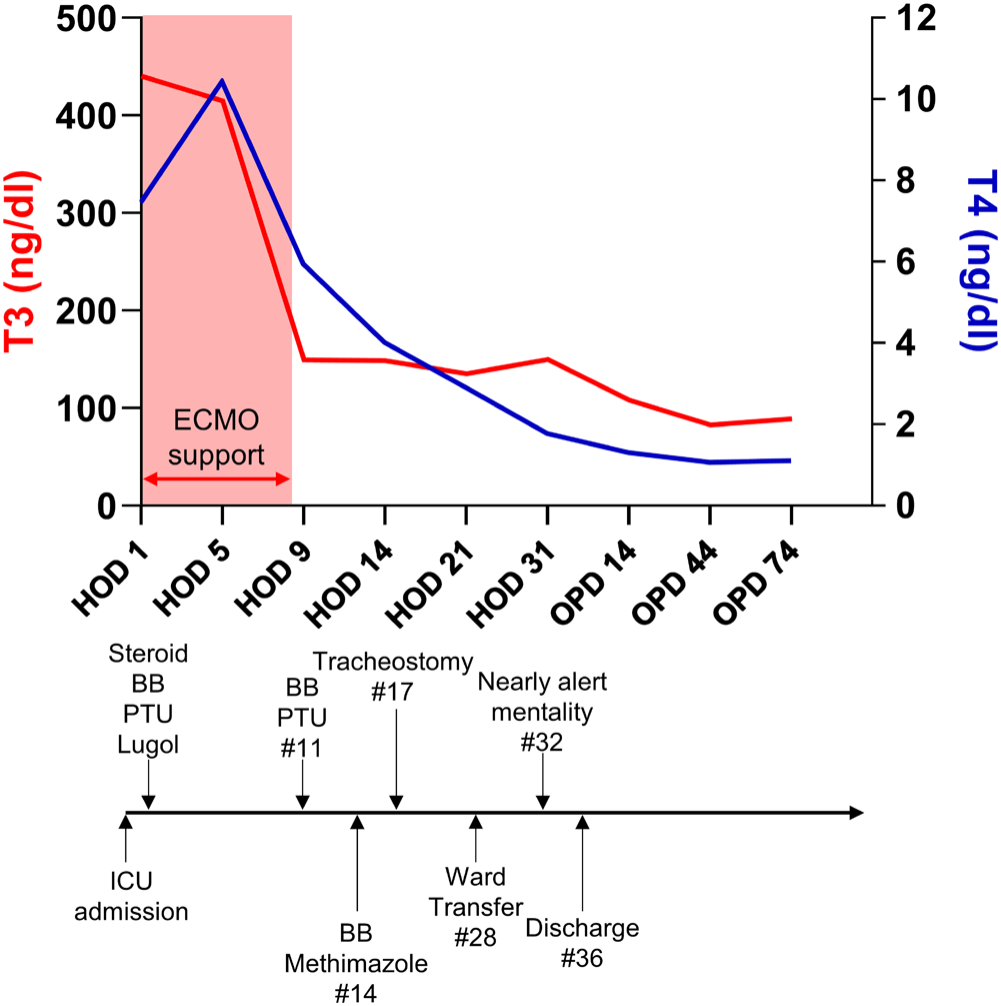
Profile of triiodothyronine (T3) and thyroxine (T4) levels with treatment and clinical manifestation. BB = beta blocker, HOD = hospital day, ICU = intensive care unit, OPD = out patient department, PTU = propylthiouracil.

**Figure 2.**
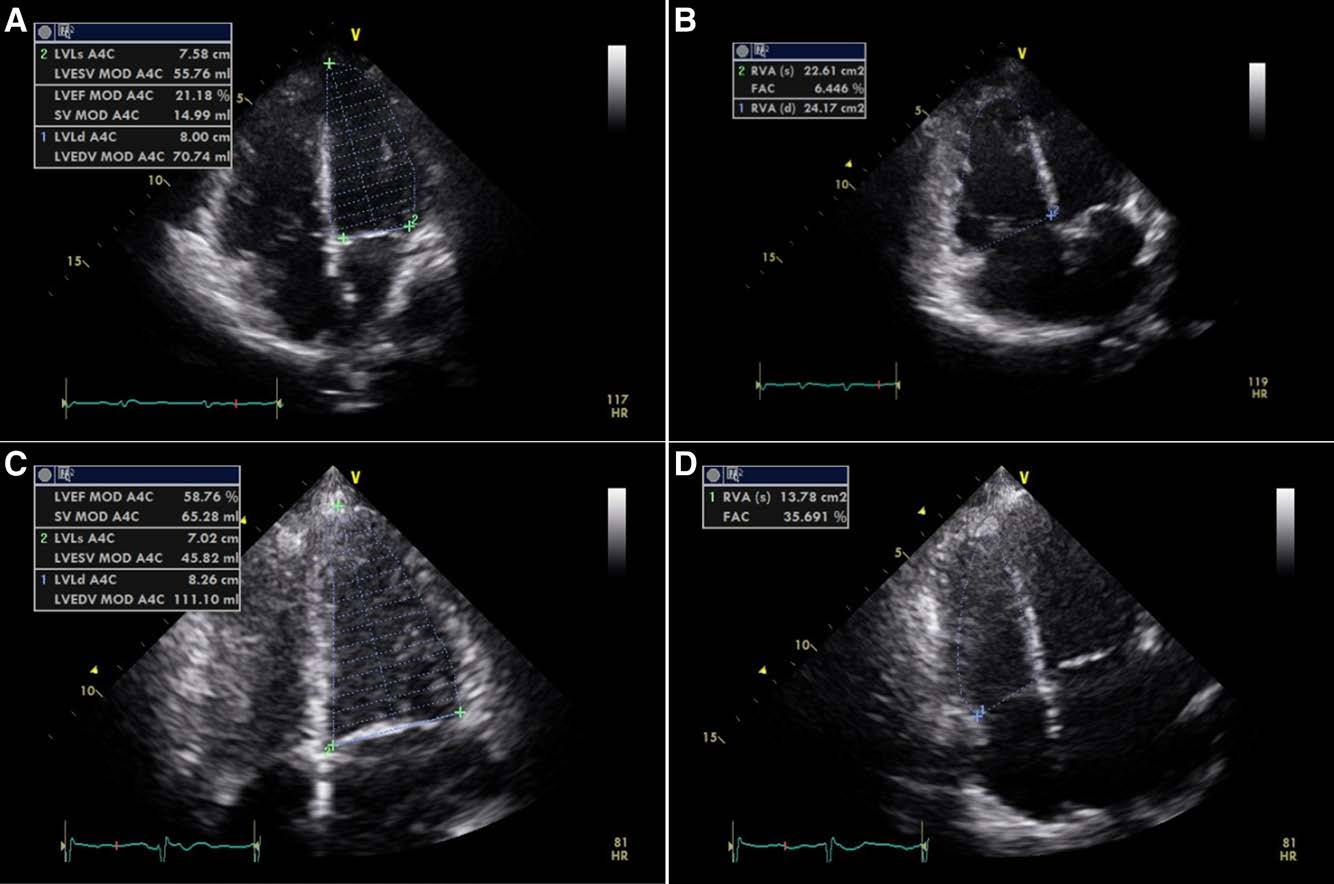
Ejection fraction of left ventricle and changes in fractional area of the right ventricle on the ECMO start and wean date. LV function and RV function of patient by echocardiography on days 2 and 8 of ECMO support. On day 2, RV dysfunction with an FAC of 6% and global hypokinesia was observed to have an overall decrease in LV function with an EF of 28%. On day 8 after ECMO support, the LV function improved (EF 60%), and the initially observed decrease in RV contractility also showed improvement (FAC 35%) in the F/U echocardiogram. ECMO = extracorporal membrane oxygenation, EF = ejection fraction, FAC = fractional area change, LV = left ventricular, RV = right ventricular.

A 67-year-old woman, who had no specific medical history, was admitted to the local hospital after a pedestrian traffic accident. At first, her vital sign was stable; however, she was transferred to Ajou University, level 1 trauma center after intubation, due to a sudden onset of compromised mental status and desaturation. No specific findings were observed in the initial brain computed tomography (CT) scans performed at the local hospital. However, a newly developed minimal falx hemorrhage was observed in a brain CT performed immediately after transfer to the hospital. Abdomen-pelvis CT scans with contrast showed stable pelvic fractures, with retroperitoneal hematoma but no evidence of active bleeding. Although cardiac enzyme levels were elevated, no ischemic signs were observed in an electrocardiogram (ECG). Despite being admitted to an intensive care unit and receiving sufficient volume replacement and inotropic support (0.381 µg/kg/min of norepinephrine and 0.13 U/min of vasopressin), the patient continued to be in shock. Atrial fibrillation with a rapid ventricular response was observed via ECG, followed by a gradual decrease in blood pressure and ultimately cardiac arrest. Return of spontaneous circulation was achieved after 1 cycle of cardiopulmonary resuscitation; however, ECMO was performed due to persistent unstable vital signs and finding of right ventricle dysfunction in echocardiogram. No specific left ventricle findings were observed with an ejection fraction (EF) of 50% without wall motion abnormality; however, a decrease in right ventricular (RV) contractility with high RV systolic pressure (46 mm Hg) was observed (fractional area change [FAC], 23%). No pulmonary thromboembolism was observed in pulmonary angio-CT performed to exclude factors that could cause RV failure. After ECMO support, metabolic acidosis that continued with shock was corrected, with the vital signs stabilized. ECG showed a normal sinus rhythm, but atrial fibrillation was repeatedly observed. In an electroencephalography(EEG) performed to evaluate the initial seizure event, findings suggestive of severe diffuse cerebral dysfunction with a “burst” attenuation pattern were observed. In the blood test performed to differentiate the cause of acute heart failure and change of consciousness, hyperthyroidism was identified with triiodothyronine (T3) levels above the upper high range (440 ng/dL, normal range: 60–181), free thyroxine (T4) of 7.45 ng/dL (normal range: 0.89–1.76), and thyroid stimulating hormone (TSH) < 0.008 µIU/mL (normal range: 0.55–4.78) (Fig. 1). The patient had a Burch and Wartofsky score of 80, well beyond the criteria for diagnosis of a thyroid storm (>45 points) (Table 1) (those that correspond to the patient’s symptoms are indicated by underlined and bold). IV glucocorticoid (200 mg QD) and PO propranolol (20 mg bid), propylthiouracil (200 mg, 6 times), and Lugol solution (0.5 g qid) were initiated. On day 2 of ECMO support, echocardiography showed RV dysfunction with an FAC of 6%, and global hypokinesia was observed with an overall decrease in left ventricular (LV) function with an EF of 28% (Fig. 2). On day 5 of hospital day (HOD), hyperthyroidism similar to that of initial results was observed in the F/U lab (T3: 414 ng/dL, free T4: 10.43 ng/dL, and TSH: <0.008 µIU/mL TSH). On day 8 after ECMO support, ECMO weaning was performed, as the LV function improved (EF 60%), along with the improved RV contractility (FAC 35%) in the F/U echocardiogram. On day 9 of HOD, in the F/U lab, hyperthyroidism was still observed, but the trend was improving (T3: 149.3 ng/dL, free T4: 5.94 ng/dL, TSH: <0.008 µIU/mL). On day 11 of HOD, glucocorticoid was tapered out, propylthiouracil 150 mg was reduced to tid, Lugol solution was stopped. On the third day after ECMO weaning (day 10 of HOD), echocardiography showed no RV dysfunction; however, decreased LV function (60% to 45%) was evident, accompanied by focal wall motion abnormality. On day 14 of HOD, we observed a T3 level of 148.7 ng/dL, a free T4 level of 4.02 ng/dL, and <0.008 µIU/mL TSH; the antithyroid agent was changed to methimazole (20 mg bid). Management of the thyroid storm and cardiogenic shock had stabilized to some extent, so sedative agents were stopped on day 10 of HOD. However, the patient’s comatose status continued, brain evaluation was performed on day 16 of HOD. Continuous generalized polymorphic moderate-amplitude theta/delta activity suggestive of cerebral dysfunction were observed on her EEG, as well as numerous multifocal microbleeds in the cerebral white matter, corpus callosum, cerebellar hemisphere and pons, suggestive of diffuse axonal injury in brain magnetic resonance imaging scans. On day 17 of HOD, open reduction and internal fixation were performed for a right humerus neck fracture, and a tracheostomy was conducted due to difficult ventilator weaning given the patient’s comatose status. The patient was transferred to the general ward on day 28 of HOD, showed alert mentality on day 32, and was transferred to a nursing hospital after sealing off the tracheostomy site on day 36. Fourteen days after being discharged from the hospital, follow-up lab results showed an improvement in hyperthyroidism (T3: 108.2 ng/dL, free T4: 1.77 ng/dL, and <0.008 µIU/mL TSH); thus, the patient’s medication dose was reduced with methimazole (10 mg bid) and then reduced to 10 mg qd of methimazole after 1 month. One month after methimazole qd reduction by 10 mg, the thyroid hormone levels had stabilized (T3: 89.1 ng/dL, free T4: 1.06 ng/dL, and <0.008 µIU/mL TSH) (Fig. 1).

**Table 1:** Burch and Wartofsky scoring system for thyroid storm.

Burch and Wartofsky score		
25–44: Impending storm	>45: Thyroid storm	
Diagnostic criteria		Points
Temperature (°C)	37.2–37.7	5
	37.8–38.2	10
	38.3–38.8	15
	38.9–39.4	20
	39.5–39.9	25
	>40	30
CNS effect	Mild (agitation)	10
	Moderate (delirium/psychosis)	20
	Severe (seizure/coma)	**30**
Gastrointestinal – hepatic dysfunction	Moderate (diarrhea/nausea-vomiting/pain)	10
	Severe (jaundice)	20
Tachycardia	99–109	5
	110–119	10
	120–129	15
	130–139	20
	>140	**25**
Congestive heart failure	Mild (pedaledema)	5
	Moderate (bibasilarrales)	10
	Severe (pulmonaryedema)	**15**
Atrial fibrillation	Absent	0
	Present	**10**
Precipitant history	Negative	0
	Positive	10
		**80**

## 3. Discussion

We report here a case of cardiogenic shock with cardiac arrest caused by a posttraumatic thyroid storm in a patient with no specific history, who was successfully treated with temporary ECMO support and proper medical treatment. Cardiac arrest due to thyroid storm are very rare clinical diseases with high mortality.^[12]^ In a study of 5 cases who underwent ECMO support in thyroid storm, the mortality rate was 40%.^[13]^ Considering that previous studies have reported a 30% mortality rate for all thyrotoxicosis patients, the mortality rate of 40% in a patient with circulatory collapse renders ECMO a good treatment option.

In most cases, it occurs in people with a history of thyroid disease. In the previously mentioned study of collecting 5 thyroid storm patients who received ECMO support, 4 (90%) had hyperthyroidism before the onset of the thyroid storm. Moreover, in a previous study in the United States, 75.1% of patients had Graves disease or had not taken proper medication and/or had undergone thyroid and parathyroid-related surgery.^[3]^ In addition to hyperthyroidism, nonthyroidal surgery, infection, diabetic ketoacidosis, and acute iodine load are known triggers of this condition.^[14]^

This patient was administered a nonselective beta blocker immediately after thyrotoxicosis was suspected. In addition to beta-blockers, thioamides were used to block the action of thyroid peroxidase, an enzyme required for the synthesis of T3 and T4.^[15]^ In addition a glucocorticoid was used to inhibit the conversion of T4 to T3,^[16]^ and Lugol solution was administered to reduce thyroid iodine uptake, iodine oxidation, and organification.^[17]^ Although some reports reported that thyroidectomy was performed for complete resolution of thyrotoxicosis,^[18,19]^ in this case, the patient’s family did not want surgical treatment, so only medical treatment was maintained.

The mechanism by which thyrotoxicosis induces myocardial dysfunction is not clearly known. Hyperthyroidism decreases systemic vascular resistance while relaxing arterial smooth muscle.^[20]^ When systemic vascular resistance falls due to thyroid hormone and overlaps with tachycardia with decreasing the diastolic filling time, cardiac output is significantly reduced.^[19]^ When this happens, atrial systolic function becomes very important.^[20]^ However, in this case, atrial fibrillation with a rapid ventricular response occurred; thus, refractory cardiogenic shock ensued. In addition, T4 itself may reduce the alpha-1 receptor-mediated vasoconstrictory effect.^[14]^ Because inotropes can show reduced effectiveness in a thyroid storm, mechanical support such as ECMO is an important treatment option for cardiogenic shock.

Diagnosing a thyroid storm in patients with multiple trauma can be difficult because most trauma patients have symptoms of tachycardia, altered mental status, and abdominal pain that appear along with a thyrotoxic status. Furthermore, it is more difficult to prioritize a thyroid storm without a history of hyperthyroidism. However, as mentioned above, circulatory collapse can be induced by a thyroid storm, and it also creates a state in which inotropic agents do not work properly. Therefore, when unexplained shock without bleeding evidence occurs in patients with multiple trauma, a thyroid function test should be performed to rule out a thyroid storm. Moreover, if hyperthyroidism is suspected in a trauma patient, even if there is no specific history, it is important to consider the possibility of a thyroid storm and the need for mechanical support such as ECMO in patient with cardiogenic shock.
